# Global expression profiling of CD10 + /CD19 + pre-B lymphoblasts from Hispanic B-ALL patients correlates with comparative TARGET database analysis

**DOI:** 10.1007/s12672-022-00480-7

**Published:** 2022-04-21

**Authors:** Laura Castañeda-Partida, Rodolfo Ocadiz-Delgado, José Manuel Sánchez-López, Enrique García-Villa, José Gabriel Peñaloza-González, Martha Margarita Velázquez-Aviña, José Refugio Torres-Nava, Jorge Alfonso Martín-Trejo, Karina Solís-Labastida, Francisco Xavier Guerra-Castillo, Vilma Carolina Bekker-Méndez, Víctor Hugo Rosales-García, Dámaris Romero-Rodríguez, Raúl Mojica-Espinoza, Alfonso Mendez-Tenorio, Crystel A. Ramírez-Calzada, Elízabeth Álvarez-Ríos, Juan Manuel Mejía-Aranguré, Patricio Gariglio

**Affiliations:** 1 Laboratorio de Genética Toxicológica, Biología. Facultad de Estudios Profesionales Iztacala (FESI), Universidad Nacional Autónoma de México (UNAM), Tlalnepantla, Estado de México Mexico; 2 Laboratorio de Oncología Molecular, Departamento de Genética y Biología Molecular. Centro de Investigación y de Estudios Avanzados (Cinvestav), Ciudad de México, Mexico; 3 Laboratorio de Epigenética, Instituto Nacional de Medicina Genómica (INMEGEN), Mexico City, Mexico; 4 Servicio Oncología Pediátrica, Hospital Juárez de México (HJM), Mexico City, Mexico; 5 Servicio de Oncología, Hospital Pediátrico Moctezuma (HPM), Mexico City, Mexico; 6 Servicio de Hematología, Hospital de Pediatría. Centro Médico Nacional (CMN), “Siglo XXI” , Instituto Mexicano del Seguro Social (IMSS), Mexico City, Mexico; 7 Unidad de Investigación Médica en Inmunología e Infectología, Hospital de Infectología ‘‘Dr. Daniel Mendez Hernández’’, ‘‘La Raza’’, IMSS, Mexico City, Mexico; 8 Laboratorio de Citometría de Flujo, Laboratorios Nacionales de Servicios Experimentales, Centro de Investigación y de Estudios Avanzados (Cinvestav), Mexico City, Mexico; 9 Unidad de Citometría, Instituto Nacional de Enfermedades Respiratorias (INER), Mexico City, Mexico; 10 Unidad de Genotipificación y Análisis de Expresión, Instituto Nacional de Medicina Genómica (INMEGEN), Mexico City, Mexico; 11 Laboratorio Biotecnología y Bioinformática Genómica, Departamento de Bioquímica. Escuela Nacional de Ciencias Biológicas, Instituto Politécnico Nacional. MX, Mexico City, Mexico; 12 Unidad de Investigación Médica en Epidemiología Clínica, UMAE Hospital de Pediatría. Centro Medico Nacional (CMN) ‘‘Siglo XXI’’, Instituto Mexicano del Seguro Social (IMSS), Mexico City, Mexico; 13 Coordinación de Investigación en Salud, Instituto Mexicano del Seguro Social (IMSS), Mexico City, Mexico; 14 Facultad de Medicina, Universidad Nacional Autónoma de México, Mexico City, Mexico; 15 Laboratorio de Genómica del Cáncer, Instituto Nacional de Medicina Genómica, Mexico City, Mexico

**Keywords:** ARACNE, FACS, TARGET, Pediatric precursor B-ALL, *PIK3CG*, *SMIM10LB2*

## Abstract

Mexico City has one of the highest incidences of acute lymphoblastic leukemia (ALL) globally, with patients showing low survival, and high relapse rates. To gain more insight into the molecular features of B-ALL in Mexican children, we isolated CD10 + /CD19 + precursor B lymphoblasts from four bone marrow and nine peripheral blood samples of B-ALL patients using a fluorescence-activated cell sorting protocol. The global gene expression profile (BM vs PB) revealed 136 differentially expressed genes; 62 were upregulated (45.6%) and 74 were downregulated (54.4%). Pearson’s correlation coefficient was calculated to determine the similarity between pre-B lymphoblast populations. We selected 26 highly significant genes and validated 21 by RT-qPCR (*CNN3, STON2, CALN1, RUNX2, GADD45A, CDC45, CDC20, PLK1, AIDA, HCK, LY86, GPR65, PIK3CG, LILRB2, IL7R, TCL1A, DOCK1, HIST1H3G, PTPN14, CD72,* and *NT5E*)*.* The gene set enrichment analysis of the total expression matrix and the ingenuity pathway analysis of the 136 differentially expressed genes showed that the cell cycle was altered in the bone marrow with four overexpressed genes (*PLK1, CDC20, CDC45,* and *GADD45A*) and a low expression of *IL7R* and *PIK3CG,* which are involved in B cell differentiation. A comparative bioinformatics analysis of 15 bone marrow and 10 peripheral blood samples from Hispanic B-ALL patients collected by the TARGET program, corroborated the genes observed, except for *PIK3CG*. We conclude the Mexican and the Hispanic B-ALL patients studied present common driver alterations and histotype-specific mutations that could facilitate risk stratification and diagnostic accuracy and serve as potential therapeutic targets.

**Supplementary Information:**

The online version contains supplementary material available at 10.1007/s12672-022-00480-7.

## Introduction

Leukemia is the most common childhood malignancy globally. The treatment outcome of acute lymphoblastic leukemia (ALL) has improved steadily over the last 50 years [1]. In developed regions of the world, the 5-year survival is > 90% and the cure rate is 85%. However, in developing countries such as Mexico, Jiménez-Hernández et al. [2] observed a low survival rate (64%) and a relapse rate of 26.2%. Additionally, the Global Cancer Observatory (GLOBOCAN 2020) [3] reports that Mexico has high age-standardized incidence rates per sex (6.0 for males and 4.9 for females per 100,000). Furthermore, Mexico City has one of the highest ALL incidences in the world (5.76 per 100,000), similar to that of Hispanics living in the USA [4]. Therefore, in addition to the classification of risk in new cases diagnosed with ALL using clinical, cytogenetic, immunological, and molecular variables, several studies on the molecular oncology of ALL and the mestizo genetic diversity in Mexico have been conducted to determine the complex and heterogeneous clinical and biological features of this hematological malignancy in Mexican children [5], who also have a poor response to conventional therapy. Thus, in order to gain further insight into the molecular features of pediatric B-ALL in this group and considering that most of the gene expression profiles of B-ALL have been conducted using mononuclear cells (lymphocytes B and T and monocytes) from patient samples, we performed a global gene expression analysis of a sample of flow-sorted CD10 + /CD19 + precursor B (pre-B) lymphoblasts from peripheral blood (PB) or bone marrow (BM) samples of B-ALL patients, to prevent contamination with other mononuclear or non-leukemic cells. As Staal et al. have reported, the choice of technique and purification influence the identification of potential diagnostic markers [6] so this strategy allowed us to obtain more accurate data from both leukemic pre-B lymphoblast populations on the context of the disease. Bioinformatics tools were used to determine the cellular processes and signaling pathways involved in the differentially expressed genes (DEGs) of these leukemic cells. Additionally, in order to compare and corroborate our results and taking into account that Mexican children have 85% of Hispanic descent, a comparative bioinformatics analysis was performed using data from BM and PB samples of Hispanic B-ALL patients collected from the TARGET public database. The results allowed us to draw sound conclusions about the similarities and differences between both groups of patients and underlies once more the importance of the PI3K/Akt/mTOR pathway in B-ALL and points out specific cell cycle proteins as therapeutic targets for this pediatric malignancy.

## Materials and methods

### Patient samples

This study included 11 pediatric patients (< 16 years old) newly diagnosed with pre-B ALL by examining the morphology of and immunophenotyping cells from PB samples or BM aspirates. The samples were stratified into two subgroups: high risk (0–12 months or > 10 years old; leucocyte count > 50 × 10^9^/L) or standard risk (1–10 years old; leukocyte count < 50 × 10^9^/L). As it is unethical to perform bone marrow aspiration in a healthy child, it was not possible to include a normal (or healthy) bone marrow control. Thus, in this study, bone marrow was compared to peripheral blood (BM vs. PB). Additionally, the detection of chromosomal rearrangements *ETV6-RUNX1, E2A-PBX1, BCR/ABL*, and *MLL4/AF4* was conducted for each patient according to Bekker et al. [7]. Nine PB samples and four BM aspirates were collected from the patients before remission induction therapy. The study was approved by the institutional research and ethics committees (R-2011–785-064) of the Instituto Mexicano del Seguro Social (IMSS-Mexico). Written, signed, and dated informed consent was obtained from the parents/guardians of each patient in accordance with the Declaration of Helsinki.

### Extraction and purification of CD10 + /CD19 + pre-B lymphoblasts

Three to five milliliters of PB or BM from the patients were collected using Vacutainer tubes containing EDTA (BD, Franklin Lakes, NJ, USA). Immediately after, the samples were separated using Histopaque-1077 (Sigma Aldrich, St. Louis, MO, USA) and mononuclear cells were carefully collected according to the manufacturer’s instructions.

### Immunophenotyping

The extracted mononuclear cells resuspended in isotonic phosphate-buffered saline solution (PBS) were stained with Pacific Blue (PB)-conjugated anti-CD45 (HI30), phycoerythrin (PE)-conjugated anti-CD34 (561), fluorescein isothiocyanate (FITC)-conjugated anti-CD10 (HI10a), and allophycocyanin (APC)-conjugated anti-CD19 (HIB19) (Biolegend, San Diego, CA, USA). They were incubated for 30 min at 4 °C and then analyzed and purified by fluorescence-activated cell sorting (FACS) using a FACSAria II flow cytometer (BD Biosciences, San Jose, CA, USA). Lymphocytes were identified by side scatter and forward scatter properties, and CD45^+^, CD10^+^, CD34^+^, and CD19^+^ were selected to finally obtain CD10 + /CD19 + pre-B lymphoblasts. The purity of all samples was 90–95%, and they were centrifuged at 5000 rpm for 3 min and washed with PBS before RNA isolation.

### RNA isolation

Total RNA was extracted using TRIzol reagent and resuspended in RNase-free water (Invitrogen, Carlsbad, CA, USA) according to the manufacturer’s instructions. Samples with an A260/280 ratio ≥ 1.8 were selected and stored at − 70 °C until use.

### Microarray Analysis

RNA integrity was evaluated using an Agilent Bioanalyzer 2100 (Agilent Technologies, Santa Clara, CA, USA). Only samples with an RNA integrity number (RIN) > 7.5 were included. HuGene-1_0-st-v1 arrays were used to compare the gene expression (BM samples vs. PB samples) of flow-sorted CD10 + /CD19 + pre-B lymphoblasts. Microarray analysis was conducted for each BM and PB sample according to the manufacturer’s protocol (Affymetrix Inc., Santa Clara, CA, USA).

### Bioinformatics analysis

Microarray data (.cel files) were analyzed with Transcriptome Analysis Console (TAC 4.0) software from Affimetrix (Fold Change ≥ 1.7 or ≤ -1.7, p-value ≤ 0.05) and *Limma* protocol and default parameters. DEG data were analyzed using Ingenuity Pathway Analysis (IPA, 3.0) (QIAGEN Inc., https://www.qiagenbioinformatics.com/products/ingenuity-pathway-analysis). Additionally, the total expression matrix from TAC was used to perform a gene set enrichment analysis (GSEA) with the Broad Institute free online tools (http://software.broadinstitute.org/gsea/index.jsp) employing gene-set as permutation type and default parameters. DEG data were used to create a heatmap with the online tool HeatMapper using the clustering method centroid linkage and distance measurement with Pearson’s correlation coefficient [8].

### Algorithm for the reconstruction of accurate cellular networks (ARACNE) analysis

An MS Excel table was prepared by summarizing all data including calculated Log2 fold-change ratios (BM samples vs. PB samples) for each gene and normalized fluorescence data. The normalized fluorescence data per sample were processed by ARACNE; the analysis consisted of calculating the mutual information (MI) between pairs of genes to identify possible interactions [9]. The ARACNE plugin available for Cytoscape v3.1.1 was used [10]. Network calculation was performed using the Aracne Complete Mode, with the variable bandwidth mutual information algorithm, data processing inequality (DPI) tolerance of 0, and six mutual information steps. Different MI threshold values were tested, from 0.6 to 0.9.

### Real time analysis

One hundred and fifty nanograms of total RNA from each PB and BM sample were reverse transcribed in a 20 µL reaction following the manufacturer’s specifications (Invitrogen, Carlsbad, CA, USA). The relative expression of 26 DEGs was performed by real-time quantitative PCR (RT-qPCR) using a 7300 Real Time PCR System and 96-well optical reaction plates (Applied Biosystems, CA, USA)] and the oligonucleotide primers of *AIDA, CALN1, CDC20, CDC45, CNN3, GADD45A, GPR65, HCK, IL7R, LILRB2, LY86, PIK3CG, PLK1, RUNX2, STON2, TCL1A, CD72, DOCK1, HIST1H3G, NT5E, PTPN14, CCNB2, CD86, HDAC9, HIST1H2B1,* and *HIST1H3F* (Prime Time® IDT, San Diego, CA, USA). The amplification of each template was performed in triplicate in one PCR run. The data were analyzed using the Livak method: Amount of target = 2^−ΔΔCT^. The quantification of β2-microglobulin mRNA was performed as an endogenous control.

### Comparative bioinformatics analysis of Hispanic B-ALL patient samples from the public Therapeutically Applicable Research to Generate Effective Treatments database (TARGET)

To corroborate the experimental results of this expression profiling, a second bioinformatics analysis was performed using data from a public database. Using the cBio-Portal platform [11–13], we downloaded sequence data (RNAseq-FPKMs) from pediatric patients with B-ALL collected as part of the TARGET program of the National Cancer Institute (NIH) Office of Cancer Genomics (OCG) [14, 15]. The data were selected according to the following inclusion criteria: patients under 16 years old, Hispanic race, cell of origin: B precursor (pre-B), collected from either BM or PB at diagnosis, and before remission induction therapy; only a total of 25 samples met the inclusion criteria (15 BMs and 10 PBs). The data were compared (BM samples vs. PB samples) through a differential expression analysis (DEA) using the IDEAMEX. iDEP.92 tools and heatmaps, enrichment analysis, and signaling pathway analysis were performed using the iDEP.92, ShinyGO v0.66, Pathview, and GSEA tools. The significance value was p ≤ 0.05; the rest of the values were used by default. The KEGG and Gene Ontology (GO) databases were used for all analyses [16–20].

## Results

A FACS protocol was implemented to purify CD10 + /CD19 + pre-B lymphoblasts from the BM or PB of pediatric pre-B ALL patients. Strikingly, total RNA concentrations from less than 4 × 10^6^ CD10 + /CD19 + pre-B lymphoblasts could not be detected using the NanoDrop 2000 spectrophotometer (Thermo Scientific, Waltham, MA, USA) or by the Bioanalyzer to evaluate RNA integrity. Consequently, only the BM and PB samples with at least 4 × 10^6^ or more CD10 + / CD19 + pre-B lymphoblasts purified by FACS were selected for total RNA isolation for their high concentrations, purity, and integrity for the global gene expression analysis and validation of significant genes by RT-qPCR. Therefore, after multiple assays, the final sample size was 13.

**Fig. 1:**
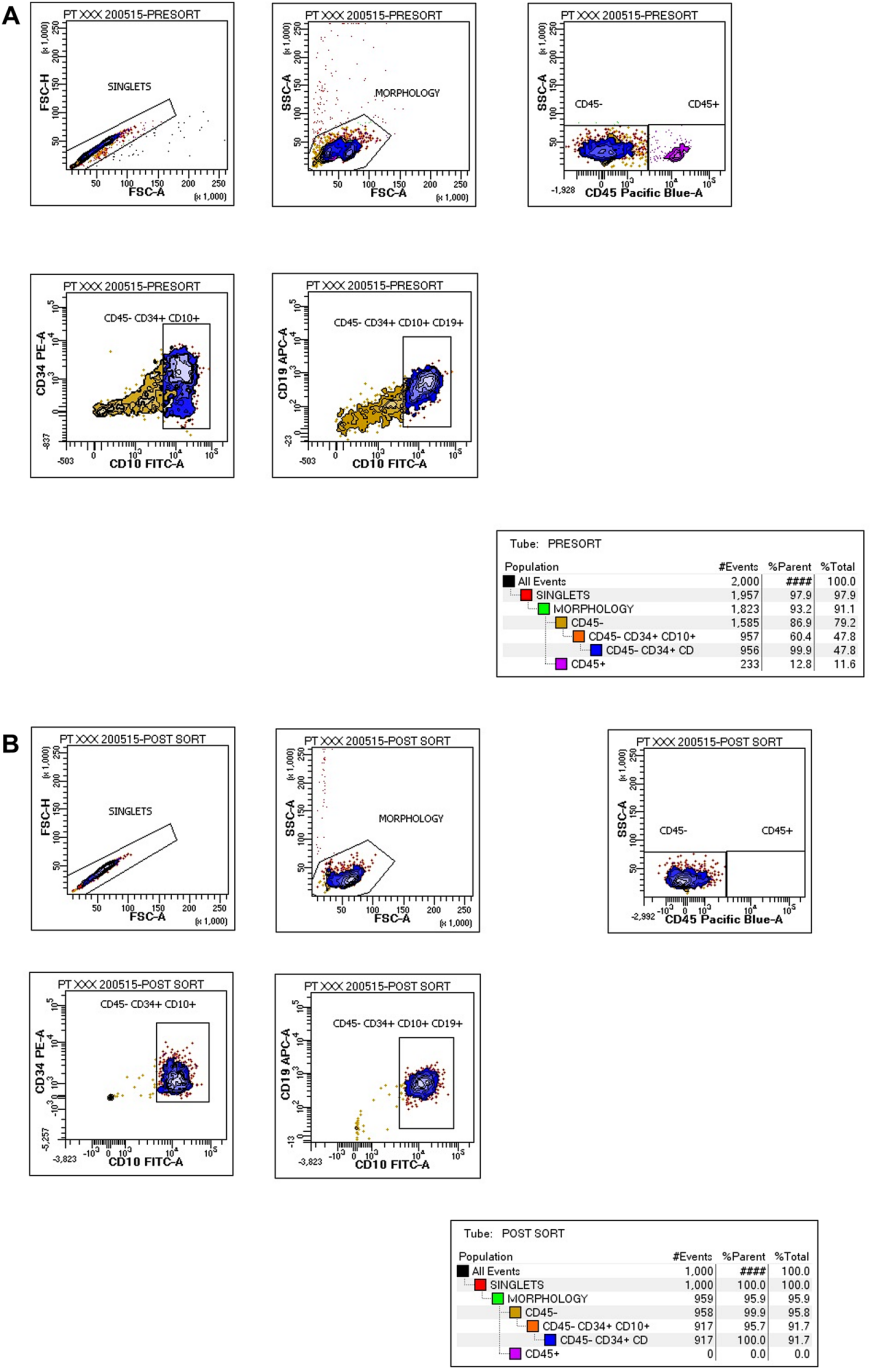
Immunophenotyping. FACS scatter plots of peripheral blood (PB) or bone marrow (BM) white phase cells from patients diagnosed with B-ALL stained with CD45-PB, CD34-PE, CD10-FITC, and CD19-APC antibodies. **A** Pre-sort scatter plot showing CD45 as an anchor antibody and CD34 for leukocyte gating**. B** Post-sort scatter plot showing CD10 + /CD19 + pre-B lymphoblasts

**Fig. 2:**
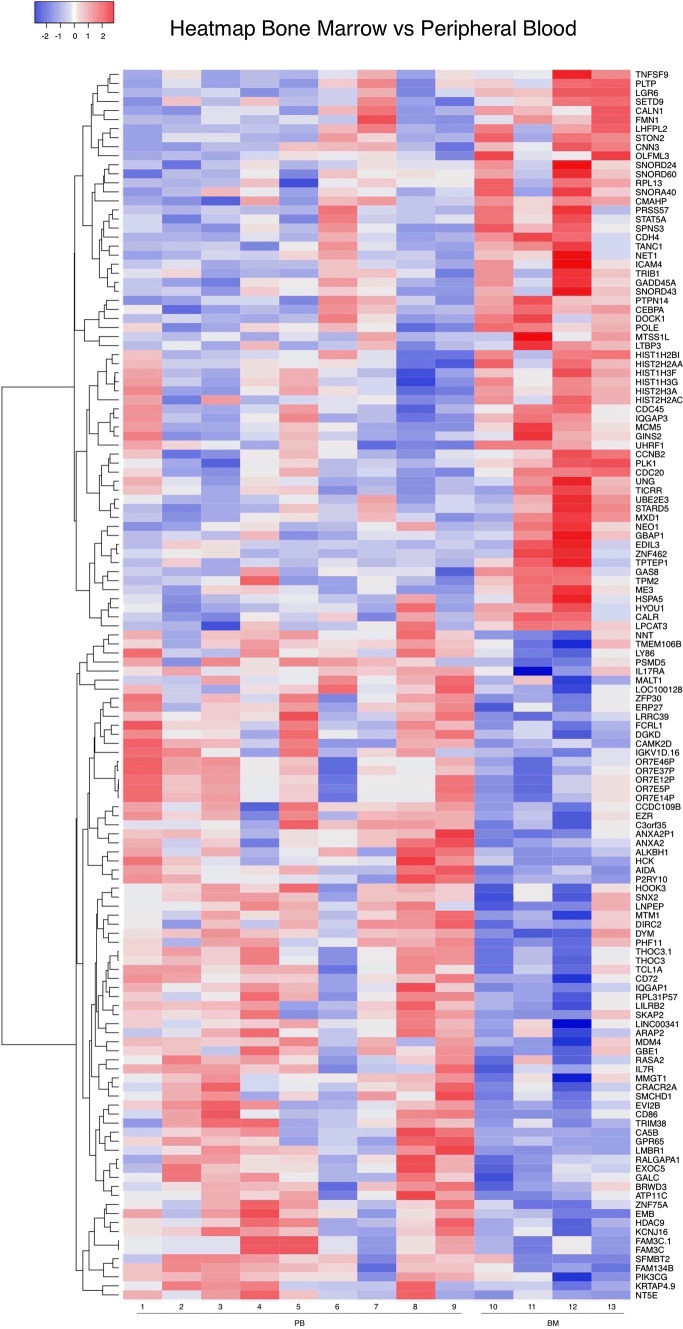
Global gene expression microarray. Heatmap of CD10 + /CD19 + pre-B lymphoblasts from peripheral blood (PB; n = 9) and bone marrow (BM; n = 4) samples of patients newly diagnosed with B-ALL. Data of the 136 differentially expressed genes (DEG): 62 upregulated (red) and 74 downregulated genes (blue) were used to create a heatmap with the HeatMapper tool using the clustering method centroid linkage and distance measurement with Pearson’s correlation coefficient

**Fig. 3:**
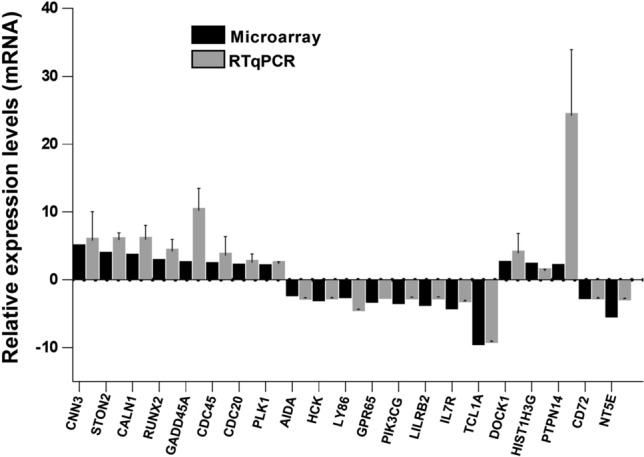
Validation of microarray data by RTqPCR. Comparison of the expression levels of selected genes determined by microarray analysis and real time PCR. The relative expression was determined by the 2^−ΔΔCT^method and the average of the values or peripheral blood (PB) samples was used as a calibrator. The RT-qPCR data are expressed as the mean/SD of three independent analyses. Black bars represent microarray values and gray bars represent RT-qPCR values

**Fig. 4:**
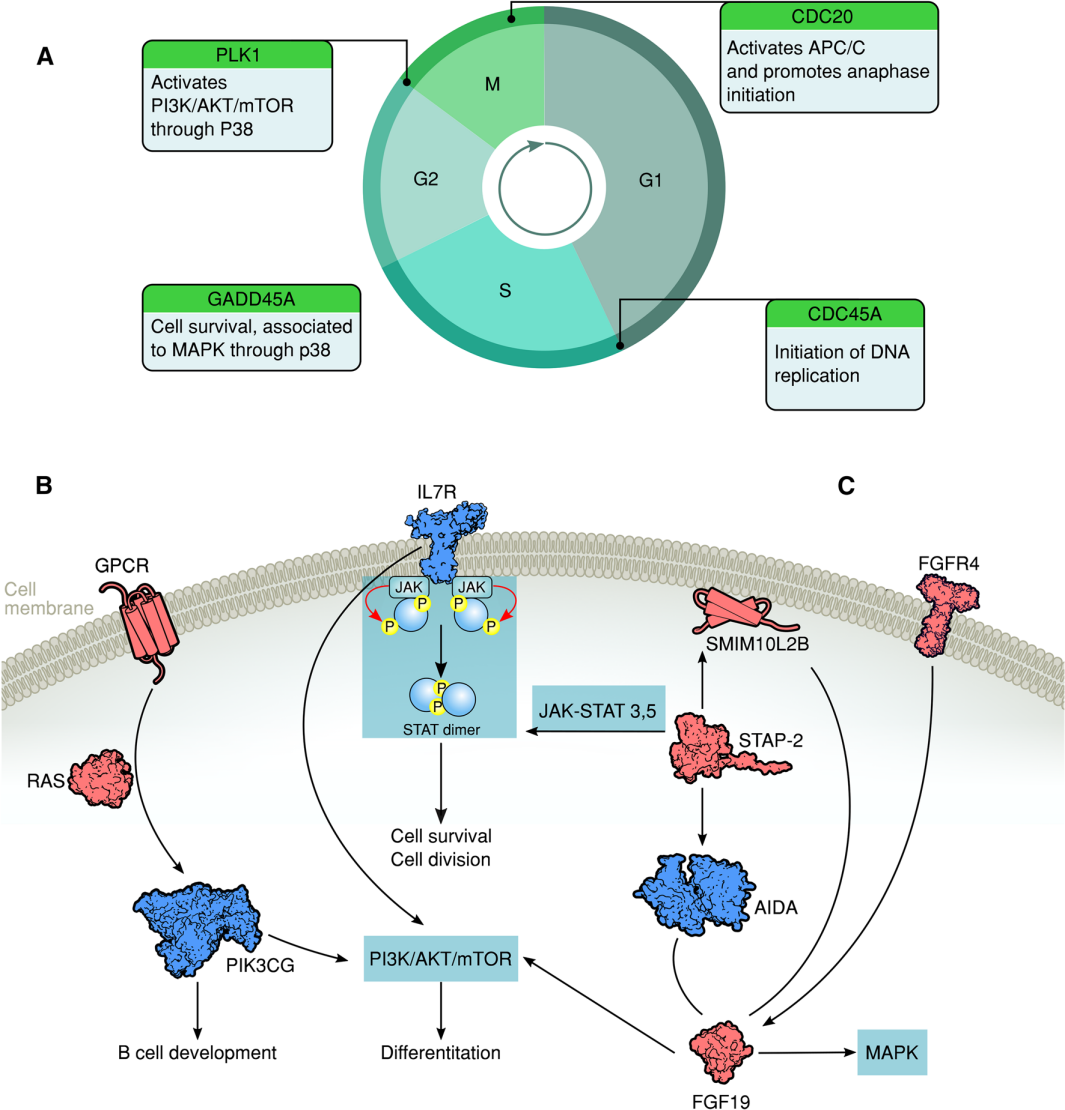
Gene signatures.** A** Upregulated cell cycle genes (*PLK1, CDC20, GADD45A*, and *CDC45)* that increase cell proliferation in flow-sorted CD10^+^/CD19^+^ pre-B lymphoblasts from BM, but not from PB samples. **B** Downregulated genes* *(IL7R* and *PIK3CG)* that inhibit differentiation or development in flow-sorted CD10^+^/CD19^+^ pre-B lymphoblasts from BM samples. **C** ARACNE inferred network for *AIDA*, a downregulated gene in this study**.** It is noteworthy that *SMIM10L2B* is an lncRNA not reported in pre B-ALL before. * In blue

Global expression profiling of CD10 + /CD19 + pre-B lymphoblasts sorted by FACS (Fig. 1) from nine PB and four BM samples from 11 patients newly diagnosed with pre-B ALL (Table 1) was performed using the Affymetrix HuGene-1_0-st-v1 chip. The hierarchical clustering analysis of data from all patients revealed 136 DEGs; 62 were upregulated (45.6%) and 74 were downregulated (54.4%). Pearson’s correlation coefficient was calculated to determine the association between the PB and BM samples and among the DEGs. The resulting heatmap, with PB samples on the left and BM samples on the right, showed differences between both CD10 + /CD19 + pre-B lymphoblast populations (Fig. 2). Some of the upregulated genes (upper half of the image) were involved in cell cycle and chromatin remodeling and were expressed in BM (red), but not in PB (blue) CD10 + /CD19 + pre-B lymphoblasts; in contrast, the downregulated genes (lower half of the image), some of which were involved in B cell differentiation, were not expressed in BM (blue) CD10 + /CD19 + pre-B lymphoblasts. Namely, BM CD10 + /CD19 + pre-B lymphoblasts proliferated and did not differentiate and PB CD10 + /CD19 + pre-B lymphoblasts did not proliferate outside as they did inside the hematopoietic niche. In line with this, and as a consequence of the methodological procedure that required high RNA concentrations that could be detected by the instruments, all BM samples showed high blast percentages (95–100%) and four PB samples (IDs 1, 4, 5, and 9) showed very high WBCs (> 50 × 10^9^/L), which corresponded to a high proliferation in the BM and subsequent release into the bloodstream. Notably, the ETV6-RUNX1 chromosomal rearrangement was detected in only one female patient (ID 12) who relapsed and eventually died. The rest of the samples did not show translocations, but a female patient (ID 1) relapsed and died. Interestingly, a male patient (ID 4) with hyperleukocytosis (WBC: 529 10^9^/L) did not relapse and has been monitored on a regular basis. The GSEA and IPA results (p-value ≤ 0.05; SF 1, SF 2 and SF 3) made it possible to further elucidate the biological cell functions and/or pathways involved and allowed the selection of the 26 top significant genes to be validated by RT-qPCR. We obtained similar results in the expression levels of 21 genes when microarray data were compared to those of RT-qPCR (Table 2; Fig. 3). In agreement with Knaack et al. [21], who performed a pan-cancer modular regulatory network analysis of six human cancers and concluded that they share a common regulatory network, we found that the validated DEGs were involved in the cell cycle (*CDC20, PLK1, GADD45A,* and *CDC45*), immune response (*TCL1A, GPR65, LILRB2, LY86, CD72,* and *NT5E*), chromatin remodeling (*HIST1H3G*), and vesicle trafficking (*STON2* and *CNN3*) and were either part of or encoded for important kinases upstream and downstream major signaling pathways for B cell differentiation (*RUNX2, PIK3CG, IL7R,* and *HCK*). Furthermore, it is noteworthy that most of these genes were found to be mainly associated with the PI3K/Akt/mTOR, MAPK, and JAK/STAT signaling pathways. On the other hand, the ARACNE analysis (MI threshold values ranging from 0.6 to 0.9) inferred a network with *AIDA* (SF 4), one of the validated genes, and three more genes: *STAP2, FGF19*, and *SMIM10L2B*, a long non-coding RNA (lncRNA). *AIDA* showed similar results at MI threshold values 0.7 and 0.8. Additionally, the ARACNE analysis (MI threshold value 0.7) inferred associations for two of the cell cycle genes observed in this study; *PLK1* was associated with *OR11H7* (SF 5)*,* an olfactory receptor, and *CDC45* was associated with *KCNQ1DN* (SF 6)*,* which is also affiliated with the lncRNA class. Figure 4 summarizes the most important observations of the global gene expression profiling.

**Table 1:** Data and clinical variables of patients newly diagnosed with B-ALL included in this study

Sample ID	Site	Patient ID	Gender	Age at Dx (months)	WBC at Dx (× 109)/ L	NCI risk group	Chromosomal rearrangement at Dx	Chromosomal rearrangement at Dx	Chromosomal rearrangement at Dx	Chromosomal rearrangement at Dx	Early relapse	Death
							*ETV6-RUNX1*	*E2A-PBX1*	*BCR/ABL*	*MLL4/AF4*		
1	PB	1	F	123	92.3	HR	ND	ND	ND	ND	YES	YES
2	PB	2	M	60	30	SR	ND	ND	ND	ND	NO	NO
3	PB	3	M	60	40	SR	ND	ND	ND	ND	NO	NO
4	PB	4	M	47	529	HR	ND	ND	ND	ND	NO	NO
5	PB	5	M	84	53.1	HR	ND	ND	ND	ND	NO	NO
6	PB	6*	M	187	10.24	HR	ND	ND	ND	ND	NO	NO
7	PB	7**	M	64	19.28	SR	ND	ND	ND	ND	NO	NO
8	PB	8	M	48	35.17	SR	ND	ND	ND	ND	NO	NO
9	PB	9	M	132	123.4	HR	ND	ND	ND	ND	NO	NO

					BLAST at Dx (%)							
10	BM	6*	M	187	98	HR	ND	ND	ND	ND	NO	NO
11	BM	10	F	156	99	HR	ND	ND	ND	ND	NO	NO
12	BM	11	F	168	100	HR	DETECTED	ND	ND	ND	YES	YES
13	BM	7**	M	64	95	SR	ND	ND	ND	ND	NO	NO

*B-ALL* B-Acute Lymphoblastic Leukemia, *Dx* Diagnosis, *WBC* White Blood Count, *NCI* National Cancer Institute, *PB* peripheral blood, *BM* bone marrow, *ND* not detected

^*^This patient provided BM and PB samples

^**^This patient provided BM and PB samples

**Table 2:** Differentially expressed genes (DEG)

*Gene symbol*	*Description*	*Microarray Fold change (*> *1.7 or* < *1.7)*	*p* ≤ *0.05*	*RT-qPCR Fold change*	*p* ≤ *0.05*
*CNN3*	Calponin 3, acidic	4.8	0.0248	5.79	0.0354
*STON2*	Stonin 2	3.72	0.0275	5.85	0.0015
*CALN1*	Calneuron 1	3.43	0.0349	6.57	0.0074
*RUNX2*	Runt-related transcription factor 2	2.65	0.048	4.16	0.0499
*DOCK1*	Dedicator of Cytokinesis 1	2.41	0.03	3.9	0.044
*GADD45A*	Growth arrest and DNA-damage-inducible, alpha	2.36	0.0361	10.17	0.0015
*CDC45*	Cell Division Cycle 45	2.23	0.05	3.6	0.0284
*HIST1H3G*	Histone cluster 1 H3 Family Member G	2.1	0.05	1.27	0.001
*CDC20*	Cell division cycle 20	1.98	0.0048	2.53	0.0283
*PLK1*	Polo-like kinase 1	1.9	0.0017	2.35	0.032
*PTPN14*	ProteinTyrosine Phosphatase, Non-Receptor Type 14	1.94	0.024	24.17	0.036
*AIDA*	Axin interactor, dorsalization associated	− 2	0.0093	− 2.49	0.0286
*HCK*	HCK proto-oncogene, Src family tyrosine kinase	− 2.06	0.0171	− 2.46	0.0444
*LY86*	Lymphocyte antigen 86	− 2.28	0.031	− 4.22	0.0333
*CD72*	Cluster of Differentiation 72	− 2.42	0.012	− 2.48	0.0452
*GPR65*	G protein-coupled receptor 65	− 2.95	0.044	− 2.37	0.001
*PIK3CG*	Phosphatidylinositol-4,5-bisphosphate 3-kinase, catalytic subunit gamma	− 3.16	0.0284	− 2.44	0.0378
*LILRB2*	Leukocyte immunoglobulin-like receptor, subfamily B, member 2	− 3.41	0.0157	− 2.47	0.024
*IL7R*	Interleukin 7 Receptor	− 3.95	0.05	− 2.86	0.0284
*NT5E*	5’-Nucleotidase Ecto	− 5.13	0.031	− 2.58	0.0303
*TCL1A*	T-cell leukemia/lymphoma 1A	− 9.22	0.0429	− 8.9	0.0284

Top significant upregulated and downregulated genes in flow-sorted CD10 + /CD19 + pre-B lymphoblasts from PB and BM samples of B-ALL patients and validated by RT-qPCR

*BM* Bone marrow, *PB* Peripheral blood, *B-ALL* B-Acute lymphoblastic leukemia

**Fig. 5:**
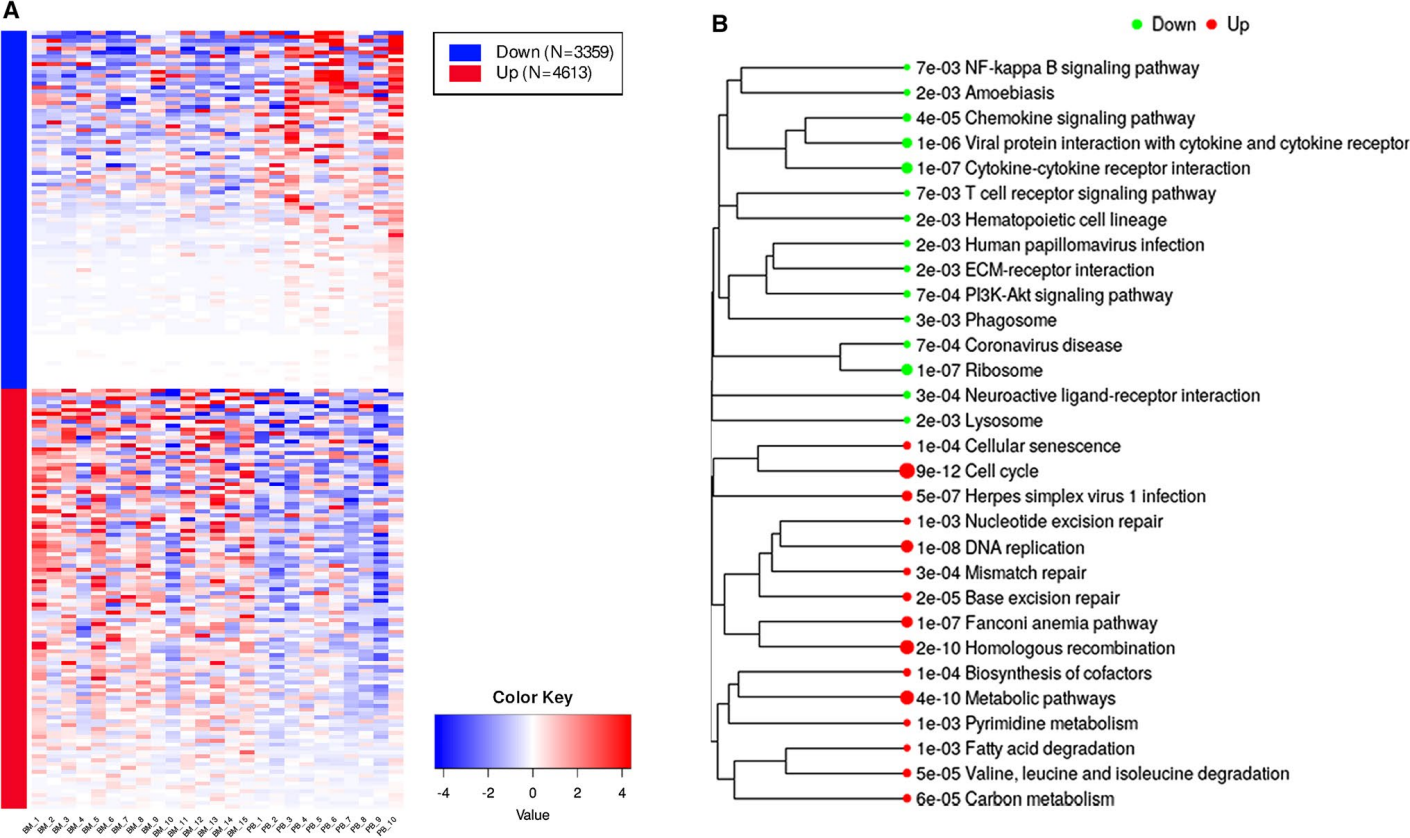
Differential expression analysis.** A** The IDEAMEX tool was used for the DEA (BM vs PB) of RNA seq data from samples of Hispanic B-ALL patients collected in the TARGET database. The resulting heatmap shows 4,316 upregulated (red) and 3,359 downregulated (blue) genes. **B** Signaling pathway cluster showing upregulated genes in the cell cycle and downregulated genes in the PI3K-Akt pathways

**Fig. 6:**
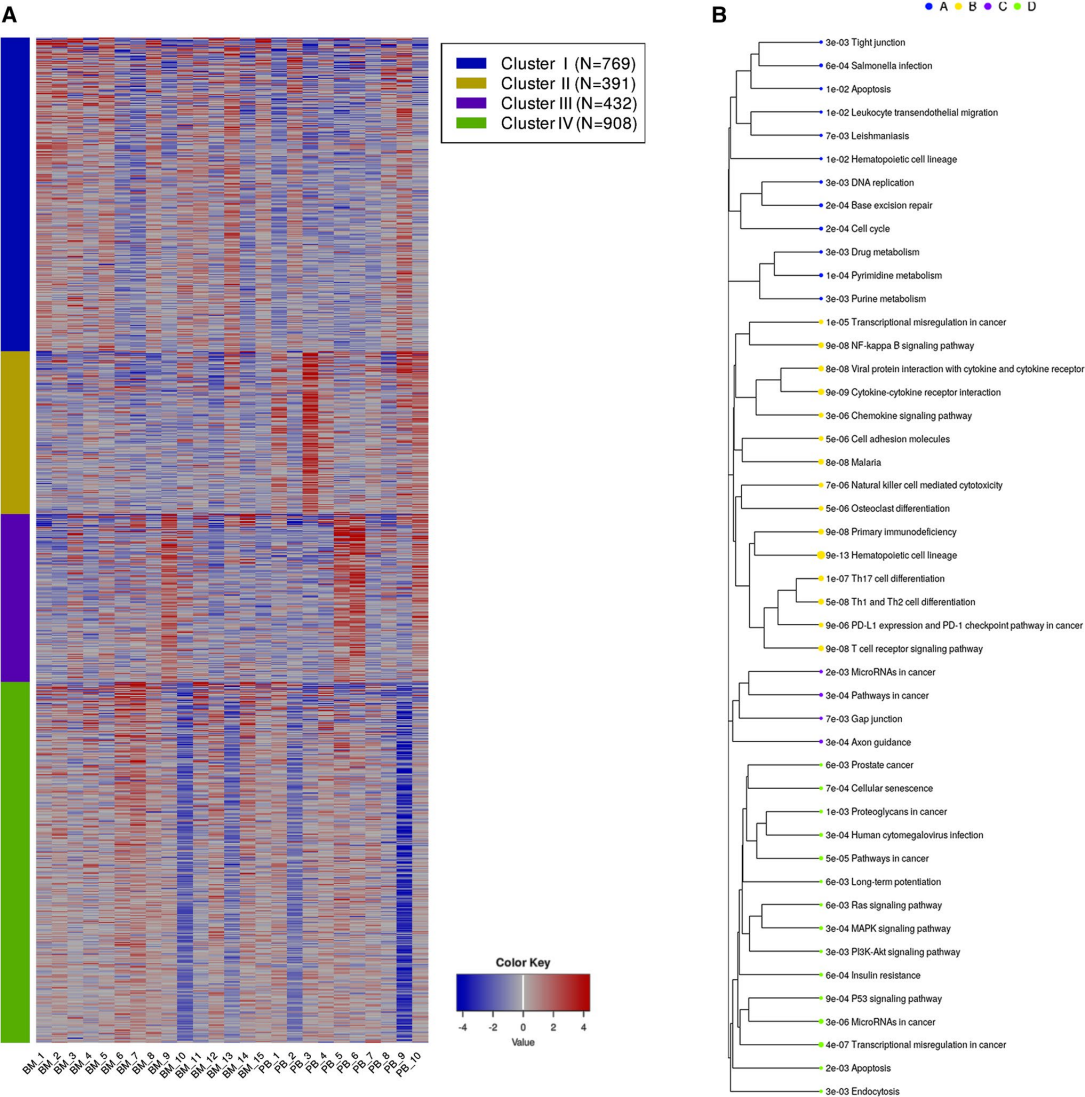
Enrichment analysis.** A** Heatmap generated through the iDEP.92 tool showing gene clusters I, II, III and IV. **B** Network cluster showing significant pathways: Cell cycle (cluster I). Hematopoietic cell linage (cluster II). Cancer pathways (cluster III). Ras 8, MAPK, and PI3k-Akt signaling pathways (cluster IV)

**Fig. 7:**
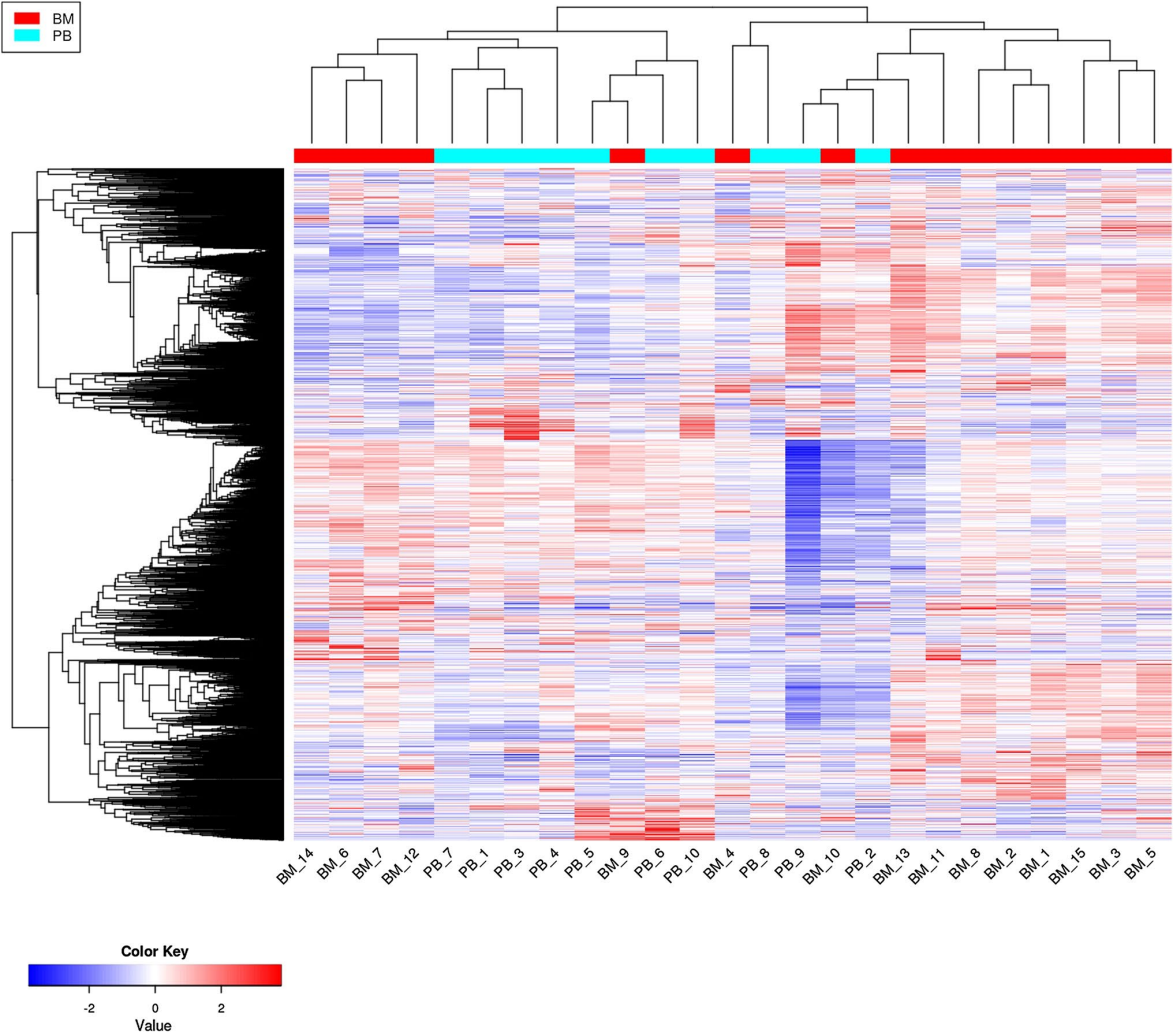
Correlation heatmap of RNA seq data of the TARGET BM vs. PB samples

**Fig. 8:**
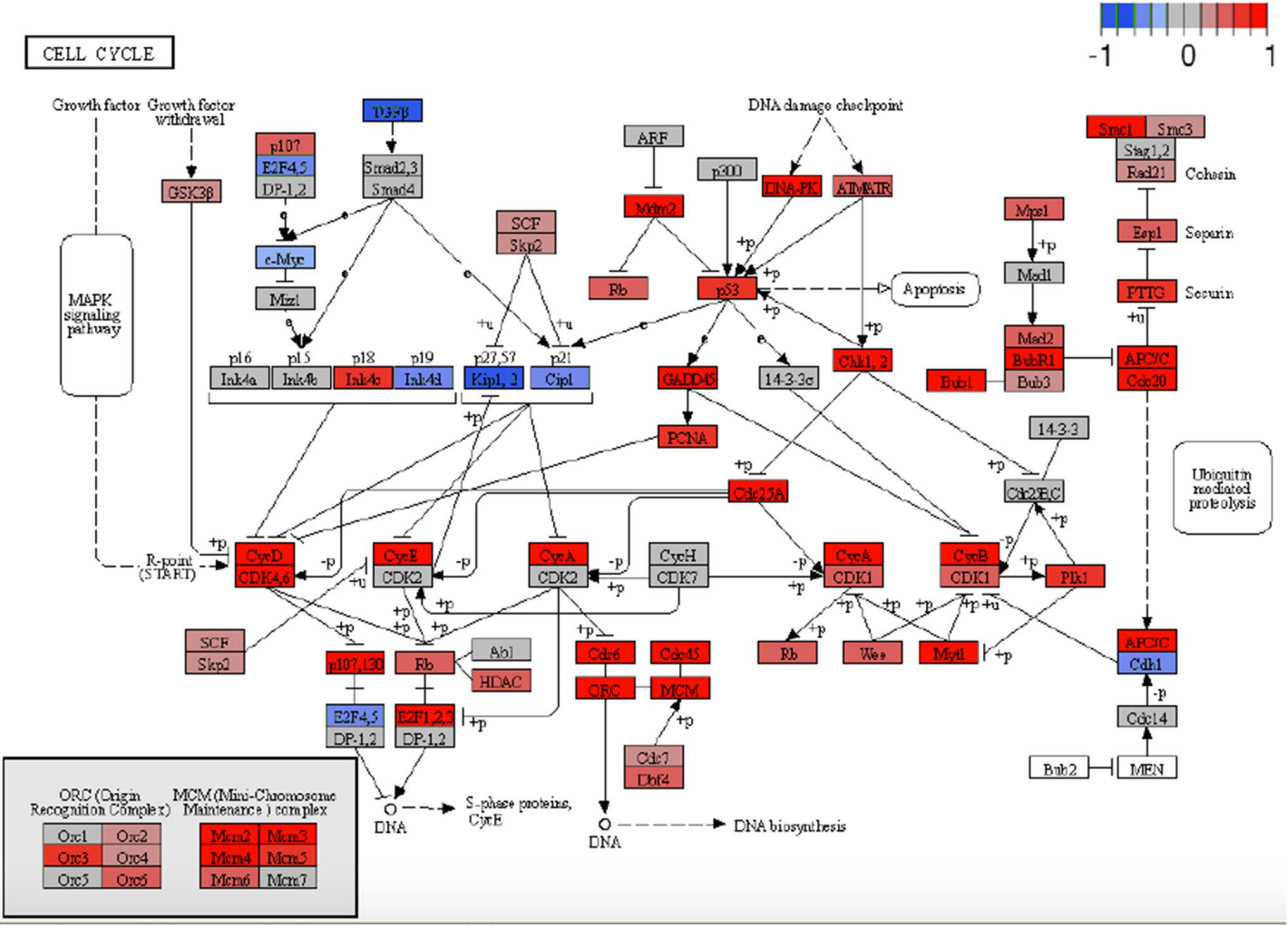
Cell cycle showing the signature of upregulated genes revealed through microarray analysis and validated by RT-qPCR (*CDC20, CDC45, PLK1,* and *GADD45A*) also overexpressed in the TARGET samples of Hispanic B-ALL pediatric patients

**Fig. 9:**
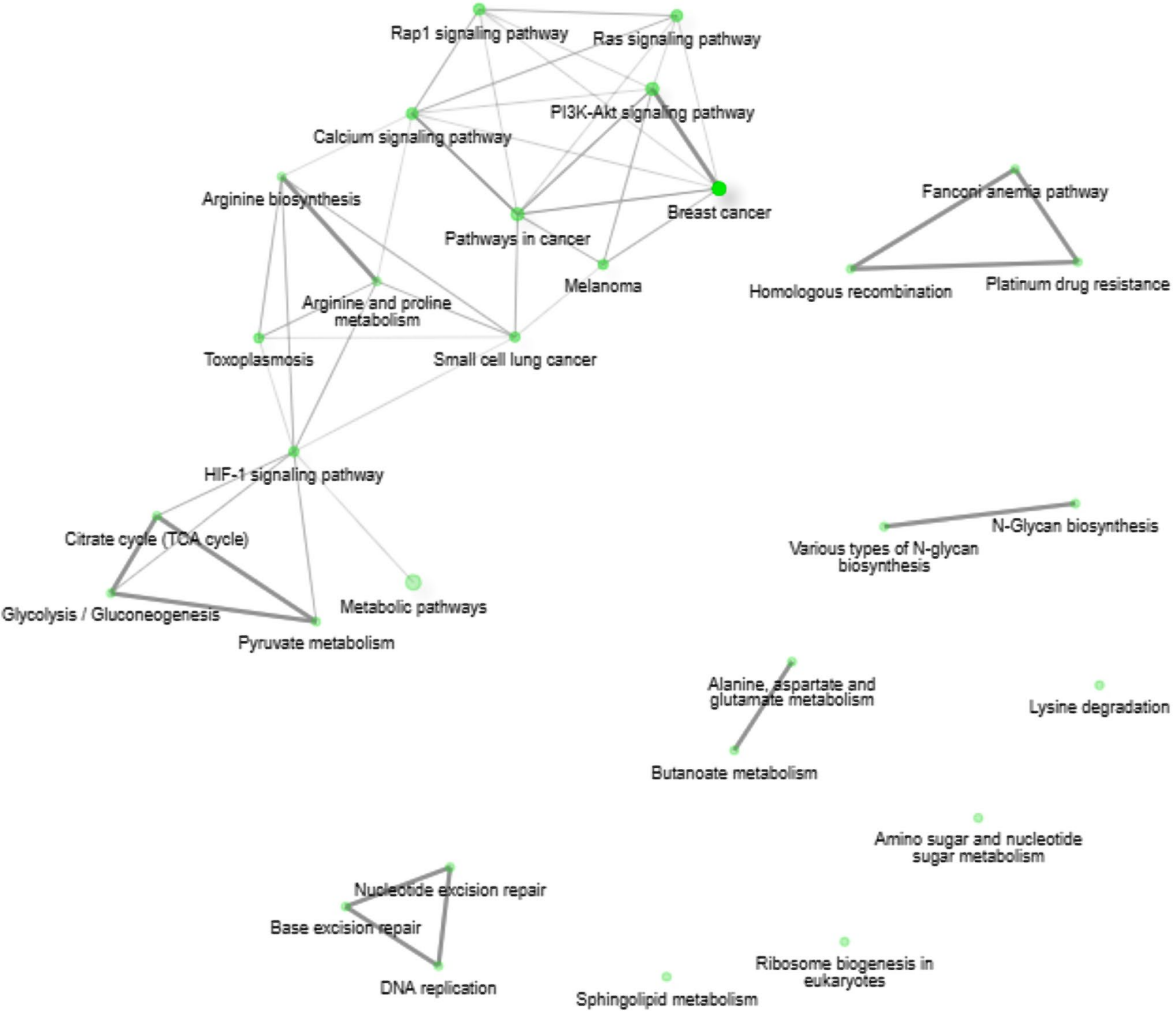
Pathway analysis. The DEG from the TARGET (BM vs PB) samples were analyzed through PATHVIEW and an enrichment analyisis was done using the KEGG database. The resulting pathway network shows the Ras and PI3k-Akt pathways, related to genes *IL7R* and *PIK3CG*

The comparative bioinformatics analysis of 25 samples (15 BM vs. 10 PB) of Hispanic pediatric patients diagnosed with B-ALL collected in the TARGET database, corroborated the findings of our microarray expression profile of pre-B CD10 + /CD19 + lymphoblasts of Mexican patients. The IDEAMEX tool was used for the DEA (BM vs PB) of RNA seq data (RNAseq-FPKMs), and the heatmap showed 4,316 upregulated and 3,359 downregulated genes (Fig. 5A), the signaling pathway cluster indicated that the former were mainly in the cell cycle (9e–12) and the latter in the PI3K-Akt pathway (7e–04) (Fig. 5B). The iDEP tool generated a heatmap with four gene clusters (Fig. 6A) and a network cluster that showed the cell cycle (2e–04) in cluster I, the hematopoietic cell lineage (9e–13) in cluster II, cancer pathways (3e–04) in cluster III, and the Ras 8 (6e–03), MAPK (3e–04), and PI3K-Akt (3e–03) signaling pathways in cluster IV (Fig. 6B). Figure 7 shows the correlation heatmap of the RNA-seq data of the BM vs. PB samples. Finally, using the iDEP, ShinyGO, Pahtview, and GSEA tools, we performed an enrichment and signaling pathway analysis. Figure 8 shows the cell cycle pathway where the signature of the upregulated genes, revealed through microarray analysis and validated by RT-qPCR (*CDC20, CDC45, PLK1,* and *GADD45A*), showed that these genes were overexpressed in the TARGET samples. Figure 9 shows the pathway network where the Ras and PI3K-Akt pathways were related to genes *IL7R* and *PIK3CG* from our gene expression profile that were downregulated.

## DISCUSSION

It is known that in ALL, B cell progenitors stop differentiating, remain immature lymphoblasts in the bone marrow, and appear in lymph nodes and the spleen after proliferation. Indeed, cell proliferation and the inhibition of apoptosis are classic events in leukaemogenesis [22]. In agreement with the GSEA results that showed an enrichment of cell cycle genes (SF 1), *PLK1, CDC20, GADD45A*, and *CDC45* ended up being overexpressed in pre-B lymphoblasts from the BM of the patients studied (Figs. 2 and 4). *PLK1* has central roles in the transition from G2 to M-phase and is considered a proto-oncogene because it activates the PI3K/Akt/mTOR signaling pathway in ALL and other hematological malignancies [23]. Its expression in leukemia cells is regulated through a PI3K- and p38-dependent pathway [24]. According to the *PLK1*-mediated cell cycle advance, it is possible that the overexpression of this gene is favoring proliferation and pre-B cell malignant transformation. Surprisingly, our ARACNE analysis inferred an association between *PLK1* and *OR11H7* (SF 5)*,* an olfactory receptor expressed in the HK-2 human proximal tubule cell line that is probably involved in the regulation of renal physiology [25]. We also observed an increase in the expression of *CDC20,* which activates the anaphase-promoting complex/cyclosome (APC/C) in the cell cycle. A high level of *CDC20* expression is significantly correlated with decreased survival in most cases of human solid tumors [26]. Herein, we reported an upregulation of *CDC20* in pre-B ALL, which may inhibit mitotic arrest and promote premature anaphase by deregulating the APC activation and resulting in genomic instability. Aneuploidy impairs proliferation in hematopoietic stem cells; among the genes involved in this process are those regulating the mitotic checkpoint, DNA damage response, and recombination, as might be the case in the pre-B ALL patients studied here. Likewise, genomic alterations may disrupt many pathways at once; for instance, Simonetti et al. [27] observed high levels of *PLK1*, an overexpression of *CDC20*, and, along with *RAD50*, a three gene signature for AML, which indicates a multistep process involving different cell cycle phases. This experimental evidence agrees with our results for pre-B ALL, as the mitotic regulators *PLK1* and *CDC20* were overexpressed in pre-B lymphoblasts from BM, suggesting that such specific genomic alterations in the cell cycle are also present in ALL and result in abnormal proliferation. The third mitotic regulator upregulated in our study was *GADD45A,* associated in vivo with signaling mediated by p38 mitogen-activated protein kinases (MAPKs) [28]. Although *GADD45A* is one of several growth arrest and pro-apoptotic genes, experimental evidence has suggested that it also functions in hematopoietic cell survival and exhibits an anti-apoptotic role. D´Angelo et al. [29] demonstrated that the in vivo constitutive activation of *GADD45A* in leukemic blasts promotes neoplastic hematopoietic cell survival, which probably occurs via p38 kinase and Bcl-xl. The overexpression of *GADD45A* in BM pre-B lymphoblasts in this study might support cell survival through simultaneous pathways according to the cellular context. The fourth mitotic regulator was *CDC45,* which is required for DNA replication. Vaisvilas et al. [30] reported a patient with a de novo 6.6 Mb duplication in a chromosomal region containing the *CDC45* gene, which resulted in pre-B ALL. They concluded that the overexpression of genes, such as *CDC45*, in the duplicated region responsible for the cell cycle, might have contributed to the formation of the leukemic clone in the bone marrow. In addition, the ARACNE analysis inferred an interaction between *CDC45* and *KCNQ1DN* (SF 6), an lncRNA related to Wilms’ tumor or nephroblastoma, the most common pediatric renal cancer affecting children 3–5 years old [31]. Furthermore, it has recently been found that *KCNQ1DN* is notably decreased in renal cell carcinoma (RCC) tissues and cell lines. It represses RCC cell growth and cell cycle progression by inhibiting c-Myc expression. *KCNQ1DN* also inhibits the transcriptional activity of the *c-Myc* promoter [32].

As with *PLK1, CDC20*, and *GADD45A*, *CDC45* showed high expression levels in the BM pre-B lymphoblasts in our analysis. Furthermore, our comparative bioinformatics analysis of data from Hispanic B-ALL pediatric patients collected from the TARGET database, corroborated the overexpression of *PLK1, CDC20*, *GADD45A*, and *CDC45* and also agrees with the results of Ma et al. [33] who conducted a pan-cancer study of pediatric cancers, including 689 B-ALL patients enrolled in the Children’s Oncology Group trials. This was expected because, as these authors found, the cell cycle is one of the 21 biological pathways disrupted by common driver alterations in pediatric cancers and because the patients in our study were indirectly selected for methodological reasons and had high WBCs. Taken together, these results could be useful in stratifying and subclassifying patients diagnosed with B-ALL and, as Simonetti et al. suggest for AML, they point out the mitotic machinery as a potential therapeutic target.

Our analysis also revealed that among the genes with low expression levels in the BM pre-B lymphoblasts, *IL7R* and *PIK3CG* (Figs. 2 and 4) could account for the inhibition of B cell differentiation. *IL7R* signaling activates the JAK/STAT signaling and PI3K/Akt/mTOR pathways, which are among the histotype-specific driver alterations in leukemias [33], and is necessary for the normal development and maintenance of both B and T cells and crucial for leukemogenesis; activating gain-of-function mutations in *IL7R* have been well described in B-ALL. However, in humans, *IL7R* inactivating mutations result in severe T-cell lymphopenia with normal, yet non-functional, numbers of B-cells [34]. In addition, the Cancer Cell Line Encyclopedia (CCLE) *IL7R* mRNA expression (RNAseq) graph shows a low value [35]. This is in agreement with our analysis, where *IL7R* exhibited almost the same low expression level reported by the *CCLE*. Although alternative pathways to activate PI3K/Akt/mTOR exist, an *IL7R* decrease would clearly impair B cell development and the proper function of these pathways. The second most significantly downregulated gene was *PIK3CG*. PI3K signaling regulates numerous biological processes, thus its deregulation results in decreased cell proliferation and increased cell death [36]. Moreover, the pharmaceutical inhibition of the PI3K/AKT pathway leads to decreased cell proliferation in pre-B ALL [37]. Nonetheless, PI3K can also be activated by G protein-coupled receptors (GPCR) through direct interaction with *PIK3CG* (catalytic PI3K isoform p110 gamma) dimers and Ras proteins, which are implicated in various aspects of immune function and regulation. Additionally, *PIK3CG* is required for early B cell development and contributes to its maintenance, proliferation, and transformation [38]. Thus, the low expression of *PIK3CG* and *IL7R* could partially account for the arrest of the normal development of B cells in BM pre-B lymphoblasts. On the other hand, it is noteworthy that resistance to anticancer chemotherapy occurs in part because the bone marrow environment promotes cell adhesion-mediated drug resistance in leukemia cells and triggers pro-survival signaling (e.g., the PI3K/AKT pathway) that allows leukemia cells to withstand chemotherapy. If *PIK3CG* is indeed involved in drug resistance in leukemia cells, it is reasonable to hypothesize that the low expression levels we observed would not generate chemotherapy resistance. Of all the patients included in this study, only two died, while the rest did not have resistant pre-B lymphoblasts and survived. Interestingly, the analysis of Hispanic B-ALL patients from the TARGET database corroborated the downregulation of *IL7R*, which is considered a common driver mutation of ALL [33, 39], but not that of *PIK3CG.* Our results showed that these B-ALL patients expressed low levels of *PIK3CA, PIK3CB,* and *PIK3CD,* which are Class I PI3Ks, such as *PIK3CG, PIK3R1*, and *PIK3R2*. Likewise, Ma et al. observed *PIK3CA* and *PIK3R1* as the most frequent mutations in the PI3K and RAS pathways in leukemias. In contrast, Gyurina et al. reported the relative expression of *PIK3CG* in bone marrow pre-B lymphoblasts from ALL and pointed out that the PIK3/AKT pathway plays a key regulatory role in BCP-ALL [33, 40].

*AIDA* was also downregulated in the expression profile analyzed in this study. This protein is localized in the cytosol, microtubules, and cytokinetic bridge. The C2 domains in AIDA proteins are important to understand the interaction between the microtubular and microfilament cytoskeleton and cellular membranes. Calmodulin is a cytoskeletal interaction domain associated with the *AIDA*-C2 domains. These findings relate *AIDA* to *CALN1*, another validated gene in this study (Table 2) that possesses C2 domains as *AIDA,* implicating their possible interaction in the cytoskeleton and vesicle trafficking functions in pre-B ALL. Surprisingly, the ARACNE analysis revealed that two of the genes associated with *AIDA* (*STAP-2* and *FGF19)* are involved in the JAK/STAT pathway that regulates cell division, survival, differentiation, and the immune system [41]. *STAP-2,* the hub of the network inferred by ARACNE, is an adaptor protein that modulates STAT3 and STAT5 transcriptional activity [42]. *STAP-2* is expressed in lymphocytes and plays a crucial role in the immune system by controlling cytokine signal transduction and modulating both innate and adaptive immune systems [43]. *FGF19,* the second gene found to interact with *AIDA,* has a high affinity for FGFR4. FGFR are required for the biological activities of FGF; the activated FGFR phosphorylate specific tyrosine residues that mediate interaction with the JAK/STAT, PI3K-AKT, and RAS-MAPK pathways [44]. Su et al. [45] observed that the levels of *FGF-19* in AML patients before chemotherapy were significantly higher than those in a control group. Although *FGF19* was not among the DEGs of our global expression analysis, thus was not validated by RT-qPCR, our ARACNE results revealed that it was involved in pre-B ALL. *SMIM10L2B,* the third gene in the ARACNE network, is a long non-coding RNA (lncRNA). lncRNAs participate in normal B-cell differentiation, but their deregulation is involved in the development of B-cell malignancies, such as ALL. lncRNA expression profiling during B-cell development has been performed in several studies that report cell-type specific expression patterns at various stages of B-cell development [46, 47]. The observation of lncRNA *SMIM10L2B* in this study is surprising, because it has not yet been reported in BM tissue or as part of the signature of lncRNAs involved in B-ALL observed in recent studies [48]. More research is needed to further understand its role and significance in this blood cancer type.

In conclusion, our global gene expression profiling of FACS-sorted CD10 + /CD19 + pre-B lymphoblasts from BM and PB and bioinformatics analysis through TAC, GSEA, and IPA revealed a four-gene signature of mitotic regulators (*PLK1, CDC20, GADD45A,* and *CDC45)* that drives the proliferation of these leukemic cells and a two-gene signature (*IL7R* and *PIK3CG)* with low expression that accounts in part for the inhibition of pre-B lymphoblast differentiation in the BM. Although the sample size of this study was small, as B-ALL patients with low WBC were not eligible for the FACS protocol we implemented, we believe that it provided pure BM and PB CD10 + /CD19 + pre-B lymphoblast samples without other contaminating white cells or non-leukemic cells. It is noteworthy that *ETV6-RUNX1*, a driver alteration of ALL [49] was the only chromosomal rearrangement detected, and only one patient eventually died. Bekker et al. [6] determined its prevalence (7.4%) in the bone marrow samples of 240 B-ALL patients and suggested that the aggressiveness of ALL in Mexican children could be due to the occurrence of this and other major gene rearrangements.

The group of four cell cycle genes observed in our global expression profile was corroborated through a comparative analysis of the TARGET database samples. Although this important pathway has many other genes (> 200), which are also overexpressed in ALL, this group seems to be important, because three of the genes have also been observed in acute myeloid leukemia (AML); herein, we reported their overexpression at different points of the cell cycle in the context of pre-B ALL. This suggests that this evidence could be used to stratify and subclassify B-ALL patients with high WBC and, as has been suggested in the case of AML, it points out these mitotic machinery proteins as potential therapeutic targets in pre-B ALL. Of the two downregulated genes, *IL7R* was corroborated by our comparative analysis, but *PIK3CG* was not. The low levels of *PIK3CG* might be a characteristic feature of Mexican pediatric B-ALL patients and underline the importance of the PI3K-mTOR pathway as a therapeutic target.

We found similarities and differences between the group studied and other Hispanic patients. The reasons for the high incidence, early relapse, and low survival rate of Mexican pediatric B-ALL patients deserve further investigation and might be explained not only by molecular oncology features but also by environmental variables.

## Supplementary Information

SF_1. PDF Gene Set Enrichment Analysis (GSEA) results (PDF 286 KB)

SF_2. TIFF Ingenuity Pathway Analysis (IPA) results (TIFF 803 KB)

SF_3. TIFF Ingenuity Pathway Analysis (IPA) results (TIFF 1801 KB)

SF_4. TIFF ARACNE network (MI 0.70) for the AIDA gene: STAP2, FGF19, and SMIM10L2B. Node colors vary from blue/green (positive log2 fold-changes, i.e., higher levels in BM samples) to orange/red (negative log2 fold-changes, i.e., higher levels in PB samples) (TIFF 1709 KB)

SF_5. TIFF ARACNE analysis results (MI threshold value 0.7) of the association between the cell cycle gene PLK1 and olfactory receptor gene OR11H7 (TIFF 2156 KB)

SF_6. TIFF ARACNE analysis results (MI threshold value 0.7) for the association between the cell cycle-related gene CDC45 and KCNQ1DN, a gene of the lncRNA class (TIFF 1378 KB)

## Data Availability

The datasets generated during the current study are available in the NCBI’s Gene Expression Omnibus [50] repository, https://www.ncbi.nlm.nih.gov/geo/query/acc.cgi?acc=GSE168593.

## References

[1] Inaba H, Mullighan CG (2020). Pediatric acute lymphoblastic leukemia. Haematologica.

[2] Jiménez-Hernández E, Jaimes-Reyes EZ, Arellano-Galindo J, García-Jiménez X, Tiznado-García HM, Dueñas-González MT (2015). Survival of Mexican children with acute lymphoblastic leukaemia under treatment with the protocol from the Dana-Farber Cancer Institute 00–01. Biomed Res Int.

[3] GLOBOCAN. March 2021. https://gco.iarc.fr/today/data/factsheets/populations/484-mexico-fact-sheets.pdf

[4] Pérez-Saldivar ML, Fajardo-Gutiérrez A, Bernáldez-Ríos R, Martínez-Avalos A, Medina-Sanson A, Espinosa-Hernández L (2011). Childhood acute leukemias are frequent in Mexico City: descriptive epidemiology. BMC Cancer.

[5] Moreno-Estrada A, Gignoux CR, Fernandez-Lopez JC, Zakharia F, Sikora M, Contreras AV (2014). The genetics of Mexico recapitulates Native American substructure and affects biomedical traits. Science.

[6] Staal FJT, van der Burg M, Wessels LFA, Barendregt BH, Baert MRM, van den Burg CMM (2003). DNA microarrays for comparison of gene expression profiles between diagnosis and relapse in precursor-B acute lymphoblastic leukemia: choice of technique and purification influence the identification of potential diagnostic markers. Leukemia.

[7] Bekker-Méndez VC, Miranda-Peralta E, Núñez-Enríquez JC, Olarte-Carrillo I, Guerra-Castillo FX, Pompa-Mera EN (2014). Prevalence of gene rearrangements in Mexican children with acute lymphoblastic leukemia: a population study—report from the Mexican interinstitutional group for the identification of the causes of childhood leukemia. Biomed Res Int.

[8] Babicki S, Arndt D, Marcu A, Liang Y, Grant JR, Maciejewski A (2016). Heatmapper: web-enabled heat mapping for all. Nucleic Acids Res.

[9] Margolin AA, Nemenman I, Basso K, Wiggins C, Stolovitzky G, Favera RD (2006). ARACNE: An algorithm for the reconstruction of gene regulatory networks in a mammalian cellular context. BMC Bioinformatics.

[10] Guitart-Pla O, Kustagi M, Rugheimer F, Califano A, Schwikowski B (2015). The Cyni framework for network inference in Cytoscape. Bioinformatics.

[11] cBio. Cancer Genomics Data Portal. https://cbio.mskcc.org/tools/cancer-genomics/index.html

[12] cBioPortal. https://www.cbioportal.org

[13] Gao J, Aksoy BA, Dogrusoz U, Dresdner G, Gross B, Sumer SO (2013). Integrative analysis of complex cancer genomics and clinical profiles using the cBioPortal. Sci Signal..

[14] National Cancer Institute Office of Cancer Genomics. Data access. https://ocg.cancer.gov/data/data-access

[15] National Cancer Institute Office of Cancer Genomics. Target: overview. 2020. https://ocg.cancer.gov/programs/target/overview

[16] Ge SX, Son EW, Yao R (2018). iDEP: an integrated web application for differential expression and pathway analysis of RNA-Seq data. BMC Bioinformatics.

[17] Jiménez-Jacinto V, Sanchez-Flores A, Vega-Alvarado L (2019). Integrative Differential Expression Analysis for Multiple EXperiments (IDEAMEX): a web server tool for integrated RNA-seq data analysis. Front Genet.

[18] Ge SX, Jung D, Yao R (2020). ShinyGO: a graphical gene-set enrichment tool for animals and plants. Bioinformatics.

[19] Luo W, Pant G, Bhavnasi YK, Blanchard SG, Brouwer C (2017). Pathview Web: userfriendly pathway visualization and data integration. Nucleic Acids Res.

[20] Subramanian A, Tamayo P, Mootha VK, Mukherjee S, Ebert EL, Gilette MA (2005). Gene set enrichment analysis: a knowledge-based approach for interpreting genome-wide expression profiles. Proc Natl Acad Sci USA.

[21] Knaack SA, Siahpirani AF, Roy S (2014). A pan-cancer modular regulatory network analysis to identify common and cancer-specific network components. Cancer Inform.

[22] Reyes-Sebastian J, Montiel-Cervantes LA, Reyes-Maldonado E, Dominguez-Lopez ML, Ortiz-Butron R, Castillo-Alvarez A (2018). Cell proliferation and inhibition of apoptosis are related to c-Kit activation in leukaemic lymphoblasts. Hematology.

[23] Goroshchuk O, Kolosenko I, Vidarsdottir L, Azimi A, Palm-Apergi C (2019). Polo-like kinases and acute leukemia. Oncogene.

[24] Kim MS, Kim GM, Choi Y-J, Kim HJ, Kim Y-J, Jin W (2013). TrkC promotes survival and growth of leukemia cells through Akt-mTOR-Dependent Up-Regulation of PLK-1 and Twist-1. Mol Cells.

[25] Kalbe B, Schlimm M, Wojcik S, Philippou S, Maberg D, Jansen F (2016). Olfactory signaling components and olfactory receptors are expressed in tubule cells of the human kidney. Arch Biochem Biophys.

[26] Wang S, Chen B, Zhu Z, Zhang L, Zeng J, Xu G (2018). CDC20 overexpression leads to poor prognosis in solid tumors. Medicine (Baltimore).

[27] Simonetti G, Padella A, Do Valle IF, Fontana MC, Fonzi E, Bruno S (2019). Aneuploid acute myeloid leukemia exhibits a signature of genomic alterations in the cell cycle and protein degradation machinery. Cancer.

[28] Salvador JM, Brown-Clay JD, Fornace AJ (2013). Gadd45 in stress signaling, cell cycle control, and apoptosis. Adv Exp Med Biol.

[29] D’Angelo V, Crisci S, Casale F, Addeo R, Giuliano M, Pota E (2009). High Erk-1 activation and Gadd45a expression as prognostic markers in high risk pediatric haemolymphoproliferative diseases. J Exp Clin Cancer Res.

[30] Vaisvilas M, Dirse V, Aleksiuniene B, Tamuliene I, Cimbalistiene L, Utkus A (2018). Acute pre-B lymphoblastic leukemia and congenital anomalies in a child with a de novo 22q11.1q11.22 duplication. Balk J Med Genet..

[31] Xin Z, Soejima H, Higashimoto K, Yatsuki H, Zhu X, Satoh Y (2000). A novel imprinted gene, KCNQ1DN, within the WT2 critical region of human chromosome 11p15.5 and its reduced expression in Wilm´s tumors. J Biochim..

[32] Yang F, Wu Q, Zhang L, Xie W, Sun X, Zhang Y (2019). The long noncoding RNA KCNQ1DN suppresses the survival of renal cell carcinoma cells through downregulating c-Myc. J Cancer.

[33] Ma X, Liu Y, Liu Y, Alexandrov L, Edmonson M, Gawad C (2018). Pan-cancer genome and transcriptome analyses of 1,699 pediatric leukemias and solid tumors. Nature.

[34] Oliveira ML, Akkapeddi P, Ribeiro D, Melão A, Barata JT (2019). IL-7R-mediated signaling in T-cell acute lymphoblastic leukemia: an update. Adv Biol Regul.

[35] Barretina J, Caponigro G, Stransky N, Venkatesan K, Margolin AA, Kim S (2012). The cancer cell line encyclopedia enables predictive modelling of anticancer drug sensitivity. Nature.

[36] Yang J, Nie J, Ma X, Wei Y, Peng Y, Wei X (2019). Targeting PI3K in cancer: mechanisms and advances in clinical trials. Mol Cancer.

[37] Sanchez V, Nichols C, Kim H, Gang E, Kim Y-M (2019). Targeting PI3K signaling in acute lymphoblastic leukemia. Int J Mol Sci.

[38] Beer-Hammer S, Zebedin E, von Holleben M, Alferink J, Reis B, Dresing P (2010). The catalytic PI3K isoforms p110γ and p110δ contribute to B cell development and maintenance, transformation, and proliferation. J Leukoc Biol.

[39] Montaño A, Forero-Castro M, Marchena-Mendoza D, Benito R, Hernández-Rivas J (2018). New challenges in targeting signaling pathways in acute lymphoblastic leukemia by NGS approaches: an update. Cancers.

[40] Gyurina K, Kárai B, Ujfalusi A, Hevessy Z, Barna G, Jáksó P (2019). Coagulation FXIII A protein expression defines three novel sub-populations in pediatric B-cell progenitor acute lymphoblastic leukemia characterized by distinct gene expression signatures. Front Oncol.

[41] Vainchenker W, Constantinescu SN (2013). JAK/STAT signaling in hematological malignancies. Oncogene.

[42] Minoguchi M, Minoguchi S, Aki D, Joo A, Yamamoto T, Yumioka T (2003). STAP-2/BKS, an adaptor/docking protein, modulates STAT3 activation in acute-phase response through Its YXXQ motif. J Biol Chem.

[43] Sekine Y (2014). Adaptor protein STAP-2 modulates cellular signaling in immune systems. Biol Pharm Bull.

[44] Ornitz DM, Itoh N (2015). The fibroblast growth factor signaling pathway. Wiley Interdiscip Rev Dev Biol.

[45] Su YZ, Wang CB, Zhou Y, Sun NT (2015). Effects of changes in serum endostatin and fibroblast growth factor 19 on the chemotherapeutic sensitivity in acute myeloid leukemia patients. Genet Mol Res.

[46] Dahl M, Kristensen L, Grønbæk K (2018). Long non-coding RNAs guide the fine-tuning of gene regulation in B-cell development and malignancy. Int J Mol Sci.

[47] James AR, Schroeder MP, Neumann M, Bastian L, Eckert C, Gökbuget N (2019). Long non-coding RNAs defining major subtypes of B cell precursor acute lymphoblastic leukemia. J Hematol Oncol.

[48] Wang W, Lyu C, Wang F, Wang C, Wu F, Li X, Gan S (2021). Identification of potential signatures and their functions for acute lymphoblastic leukemia: a study based on the Cancer Genome Atlas. Front Genet.

[49] Zaliova M, Kotrova M, Bresolin S, Stuchly J, Stary J, Hrusak O (2017). ETV6/RUNX1-like acute lymphoblastic leukemia: A novel B-cellprecursor leukemia subtype associated with the CD27/CD44 immunophenotype. Genes Chromosomes Cancer.

[50] Edgar R (2002). Gene expression omnibus: NCBI gene expression and hybridization array data repository. Nucleic Acids Res.

