# Supplementary material for: Global expression profiling of CD10 + /CD19 + pre-B lymphoblasts from Hispanic B-ALL patients correlates with comparative TARGET database analysis
Source: Discov Oncol. 2022 Apr 21;13:28. doi: 10.1007/s12672-022-00480-7 (PMC9023642; doi:10.1007/s12672-022-00480-7)

Gene Set Enrichment Analysis (GSEA) results
KEEG

**GeneSet**	**KEGG_Cell cycle**	
Normalized Enrichment Score (NES)	1.8377259	
FDR q-value	0.0223597	
**Gene symbol**	**Rank in gene list**	**Rank metric score**
1. CCNB2	8	0.5
2. GADD45A	40	0.409
3. PLK1	96	0.351
4. CDC6	228	0.294
5. CDC20	275	0.283
6. MCM5	322	0.267
7. GADD45G	366	0.257
8. CCND2	449	0.237
9. MCM4	459	0.235
10. TTK	567	0.216

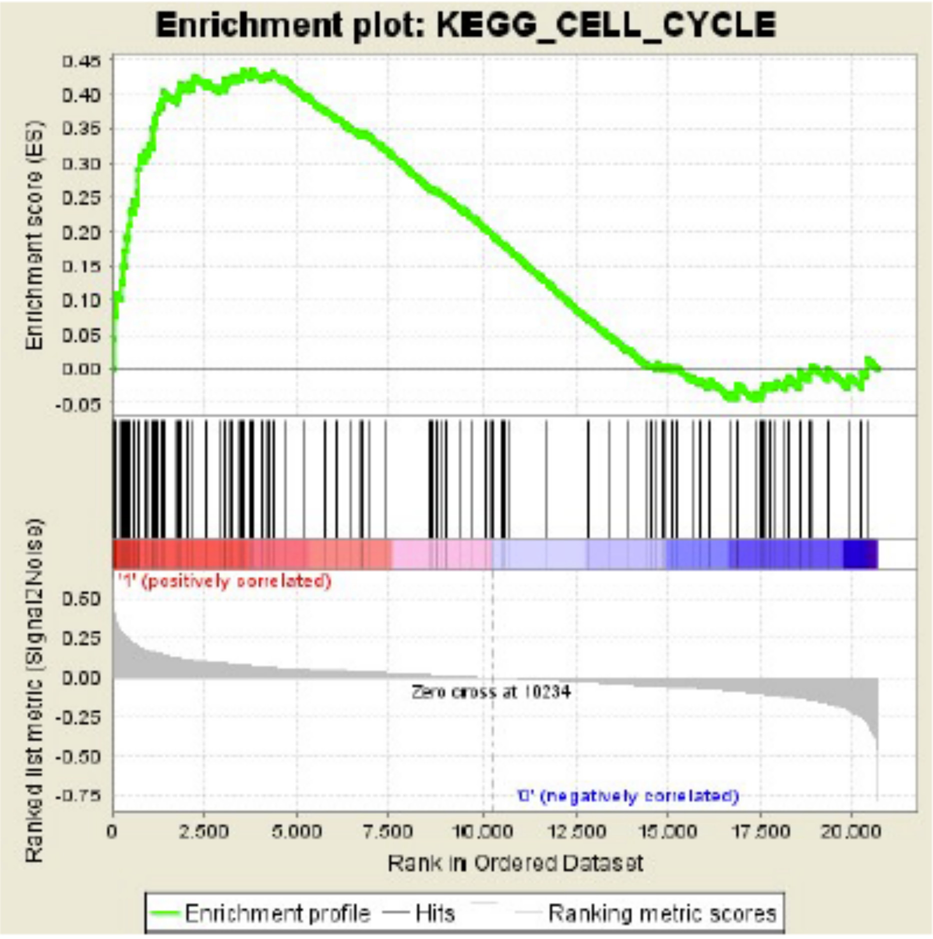

Supplement: Supplementary file 1 — SF_1. PDF Gene Set Enrichment Analysis (GSEA) results (PDF 286 KB) [file 12672_2022_480_MOESM1_ESM.pdf]
