# Supplementary figures and images for: Global expression profiling of CD10 + /CD19 + pre-B lymphoblasts from Hispanic B-ALL patients correlates with comparative TARGET database analysis
Source: Discov Oncol. 2022 Apr 21;13:28. doi: 10.1007/s12672-022-00480-7 (PMC9023642; doi:10.1007/s12672-022-00480-7)

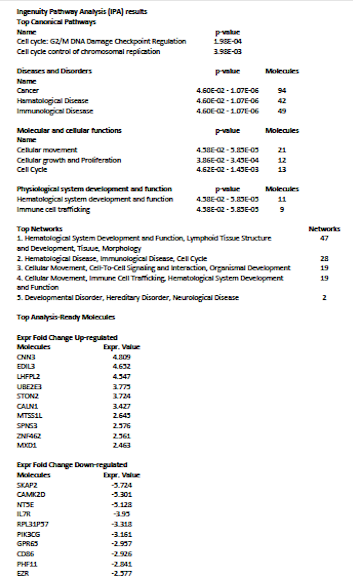

Supplement: Supplementary file 2 — SF_2. TIFF Ingenuity Pathway Analysis (IPA) results (TIFF 803 KB) [file 12672_2022_480_MOESM2_ESM.tiff]

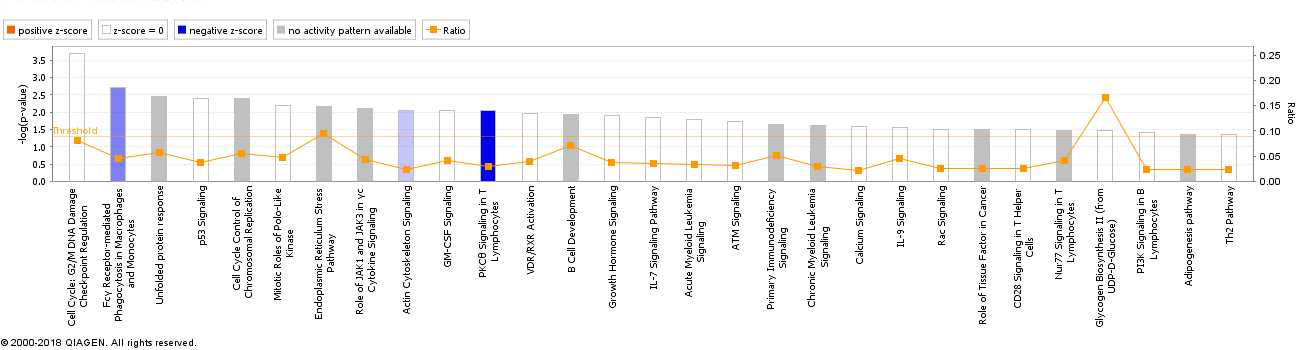

Supplement: Supplementary file 3 — SF_3. TIFF Ingenuity Pathway Analysis (IPA) results (TIFF 1801 KB) [file 12672_2022_480_MOESM3_ESM.tiff]

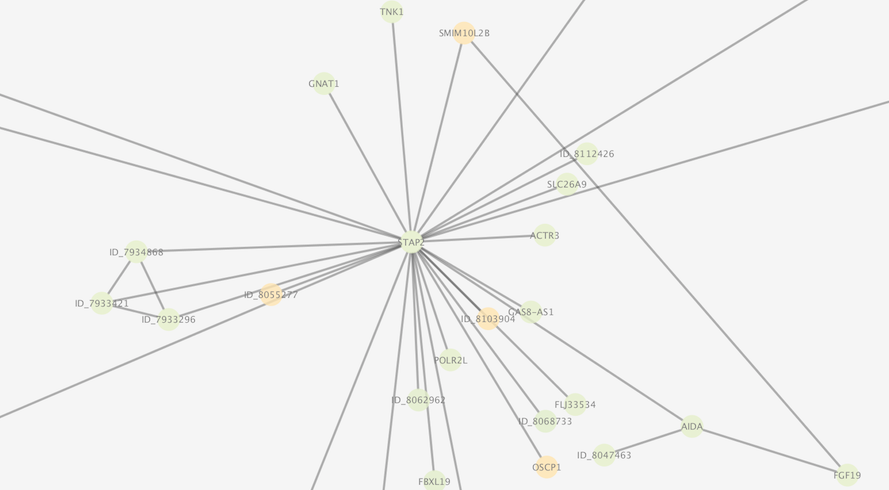

Supplement: Supplementary file 4 — SF_4. TIFF ARACNE network (MI 0.70) for the AIDA gene: STAP2, FGF19, and SMIM10L2B. Node colors vary from blue/green (positive log2 fold-changes, i.e., higher levels in BM samples) to orange/red (negative log2 fold-changes, i.e., higher levels in PB samples) (TIFF 1709 KB) [file 12672_2022_480_MOESM4_ESM.tiff]

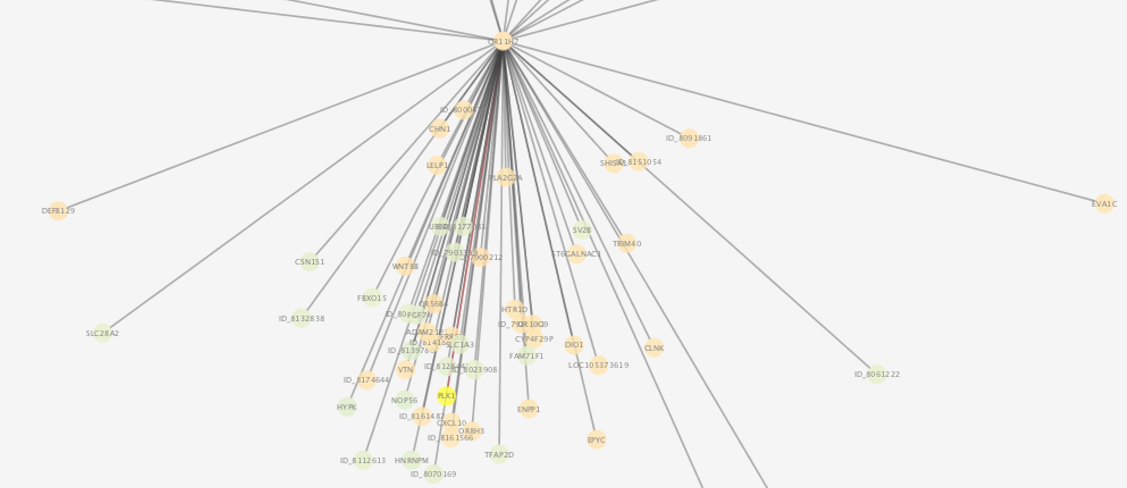

Supplement: Supplementary file 5 — SF_5. TIFF ARACNE analysis results (MI threshold value 0.7) of the association between the cell cycle gene PLK1 and olfactory receptor gene OR11H7 (TIFF 2156 KB) [file 12672_2022_480_MOESM5_ESM.tiff]

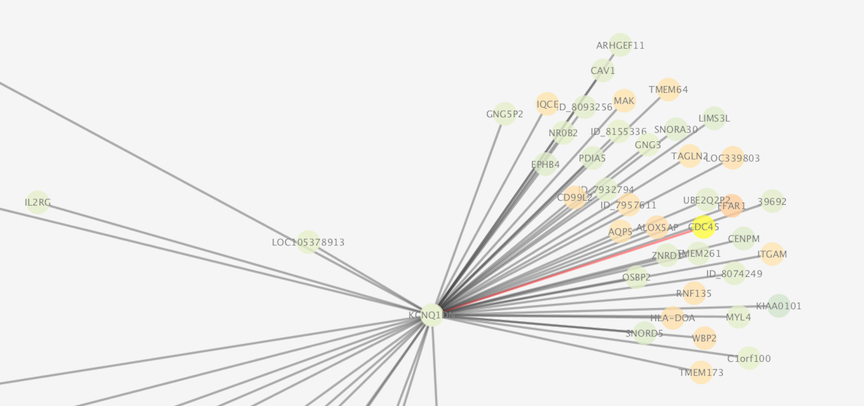

Supplement: Supplementary file 6 — SF_6. TIFF ARACNE analysis results (MI threshold value 0.7) for the association between the cell cycle-related gene CDC45 and KCNQ1DN, a gene of the lncRNA class (TIFF 1378 KB) [file 12672_2022_480_MOESM6_ESM.tiff]
